# Subtrochanteric shortening osteotomy combined with cemented total hip arthroplasty for Crowe group IV hips

**DOI:** 10.1007/s00402-013-1869-4

**Published:** 2013-10-12

**Authors:** Kenichi Oe, Hirokazu Iida, Tomohisa Nakamura, Naofumi Okamoto, Takahiko Wada

**Affiliations:** Department of Orthopaedic Surgery, Kansai Medical University, 2-3-1 Shinmachi, Hirakata, Osaka 573-1191 Japan

**Keywords:** Subtrochanteric shortening osteotomy, Cemented total hip arthroplasty, Crowe group IV, Leg lengthening, Nerve injury

## Abstract

**Background:**

Total hip arthroplasty (THA) is a challenging surgical procedure that can be used to treat severely dislocated hips. There are few reports regarding cemented THAs involving subtrochanteric shortening osteotomy (SSO), even though cemented THAs provide great advantages because the femur is generally hypoplastic with a narrow, deformed canal.

**Purposes:**

We evaluated the utility of cemented THA with SSO for Crowe group IV hips, and assessed the relationship between leg lengthening and nerve injury. Our goal was to describe surgical techniques for optimizing surgical outcomes while minimizing the risk of nerve injury.

**Methods:**

We retrospectively reviewed 34 cases of cemented THAs with transverse SSO for Crowe group IV. Prior to surgery, mean hip flexion was 93.1° (40°–130°). The mean follow-up period was 5.2 years (3–10 years).

**Results:**

Bone union took an average of 7.7 months (3–24 months). Mean leg lengthening was 40.5 mm (15–70 mm) and was greater in patients without hip flexion contracture. None of the patients experienced any nerve injuries associated with leg lengthening, and radiographic evidence of loosening was not observed at the final follow-up.

**Conclusions:**

SSO combined with cemented THA is an effective treatment for severely dislocated hips. Leg lengthening is not necessarily associated with nerve injuries, and the likelihood of this surgical complication may be related to the presence of hip flexion contracture.

## Introduction

Total hip arthroplasty (THA) for severely dislocated hips can be a very challenging procedure because it is associated with a number of technical difficulties, including lack of bone “stock” in the acetabulum (for fixing the acetabular component), a narrow femur canal, and a poorly developed abductor mechanism [1]. Placing the acetabular component at the true hip center may lead to un-coverage at the dome, necessitating a bone graft. Placing the acetabular component at the true hip center, with a bone graft, has been shown to provide successful long-term results [2]. Such outcomes from the fact that this position provides better bone coverage, improves leg length, decreases the likelihood of loosening, and reduces the resultant force acting on the hip joint during walking [3].

However, placement of the acetabular component in the true acetabulum may result in nerve injury by causing excessive leg lengthening. This can be addressed by subtrochanteric shortening osteotomy (SSO), which demands accurate anatomical knowledge and technical expertise for durable results. This procedure was first described as a treatment for congenital dislocation of the hip in older children [4], and was later applied to patients with THA [5]. Although many authors have reported SSO with un-cemented THA, there have been few reports of SSO with cemented THA [6–13]. Cemented THA has some clear advantages, such as initial fixation, ease-of-use handling of the medullary canal, and strong torsional stability. However, because the bone union may have some flaws, long-term outcomes are unpredictable.

This is a retrospective evaluation of patients undergoing combined cemented THA–SSO procedure for Crowe group IV hips. The current paper describes the technique of SSO in combination with a cemented femoral component and presents the mid-term follow-up data. It also assesses the correlation between preoperative flexion range of motion, overall lengthening and the risk for nerve injury.

## Materials and methods

### Patients

**Fig. 1:**
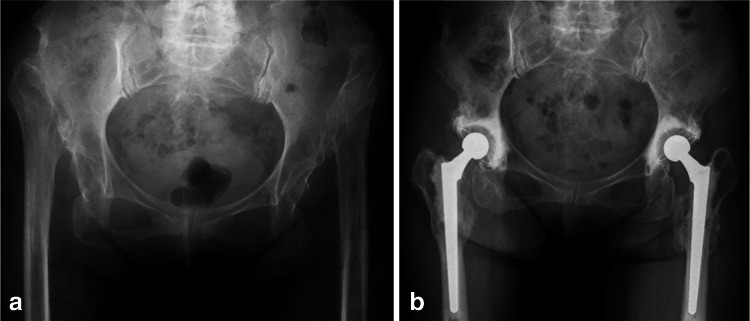
**a** A preoperative radiograph of the hips of a 75-year-old female showing bilateral dysplasia of the hip, classified as Crowe group IV. The Japanese Orthopaedic Association (*JOA*) hip score was 39 points on the right side and 63 points on the left side. **b** A radiograph taken 4 years after surgery shows bone union at the osteotomy site and no evidence of loosening. The JOA hip score was 82 points on both sides. The osteotomy site had healed by 6 months

Between November 2002 and December 2009, 34 THAs with SSO were performed in 26 patients. Patients comprised 25 females and one male with a mean age of 64.9 years (35–80 years) at the time of the operation. All of the patients suffered from dysplasia of the hip (DDH), and THAs were performed for only those patients suffering unbearable pain or a marked limp. All hips were Crowe group IV [14]; according to Charnley’s classification [15], eight patients were in Category A, 16 patients were in Category B, and two patients were in Category C. One-stage bilateral THA was performed on all of the patients in Category B; eight of these procedures required bilateral SSOs (Fig. 1). The other eight Category B patients underwent unilateral THAs with SSO. One-stage bilateral THA was carried out on all of the patients in Category C; two of these procedures required unilateral SSOs (Table 1). Eight patients had previously undergone a Schanz osteotomy.

**Table 1:** Patient characteristics

Charnley’s classification	Patients (hips)	THAs with SSO	Conventional THAs
Category A	8 (8)	8	–
Category B	16 (32)	24	8
Category C	2 (4)	2	2
Total	26 (44)	34	10

### Operative technique

**Fig. 2:**
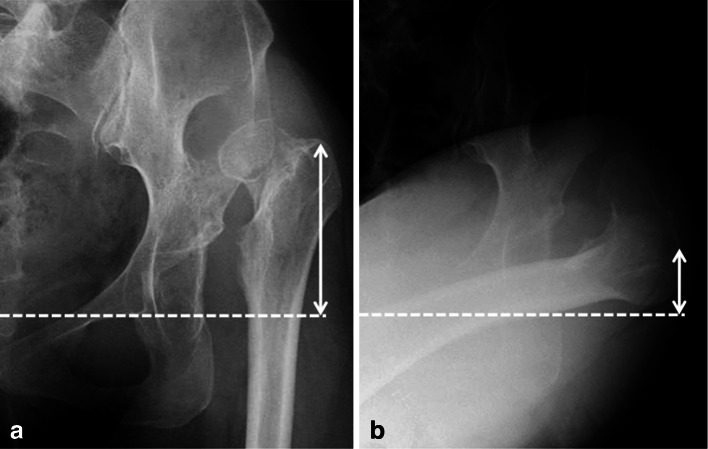
Preoperative anteroposterior radiographs showing the hip in **a** a neutral position and **b** a flexed adduction position. The *arrows* indicate the height of the greater trochanter, and the difference between these shows the expected descent of the tip of the greater trochanter. The *dotted line* indicates the teardrop line

We used Dall’s transgluteal approach to operate on the patients, each of whom was placed in the lateral decubitus position [16]. After resection of the femoral head, transverse SSO was performed according to proper preoperative planning. Anteroposterior (AP) radiographs were preoperatively taken in both neutral and flexed adduction hip positions (Fig. 2). This allowed evaluation of the expected descending length of the tip of the greater trochanter. The expected leg lengthening took into account the probable consequences for the contralateral hip in the neutral position of the AP radiographs. The anticipated amount of the femoral resection was calculated by subtracting the expected leg lengthening from the expected descent of the tip of the greater trochanter. To provide stability, we performed the femoral osteotomy below the proximal third of the expected femoral stem. We could also perform SSO later in cases where the femoral bone did not interfere with the exposure of the acetabulum. The joint capsule was partially or totally excised depending on each case to provide a clear view of the hypoplastic acetabulum; however, in order to facilitate good postoperative function, no routine abductor, iliopsoas, or external rotator release was performed.

On preoperative AP radiographs and computed tomography scans, the acetabular components were positioned in the true acetabulum. To achieve satisfactory coverage of the superior and posterior acetabulum in all patients, the roof was augmented using a structural autograft from the resected femoral head. If the femoral head was too small, we used the resected femoral shaft, after the osteotomy was complete. The structural autograft bone was fixed using poly-l-lactic acid screws (Takiron, Tokyo, Japan), as described by Wolfgang [17]. The socket was placed in its original anatomic location with 45° of abduction and 10° of anteversion. A K-MAX CLHO flanged cup (KYOCERA Medical, Osaka, Japan), which had an all-polyethylene acetabular component, was used with ENDURANCE Bone Cement (DePuy International, Leeds, UK). The diameter of the acetabular component was 38 mm in 29 hips and 40 mm in 5 hips; for all hips, the diameter of the modular head (KYOCERA Medical) was 22.225 mm.

**Fig. 3:**
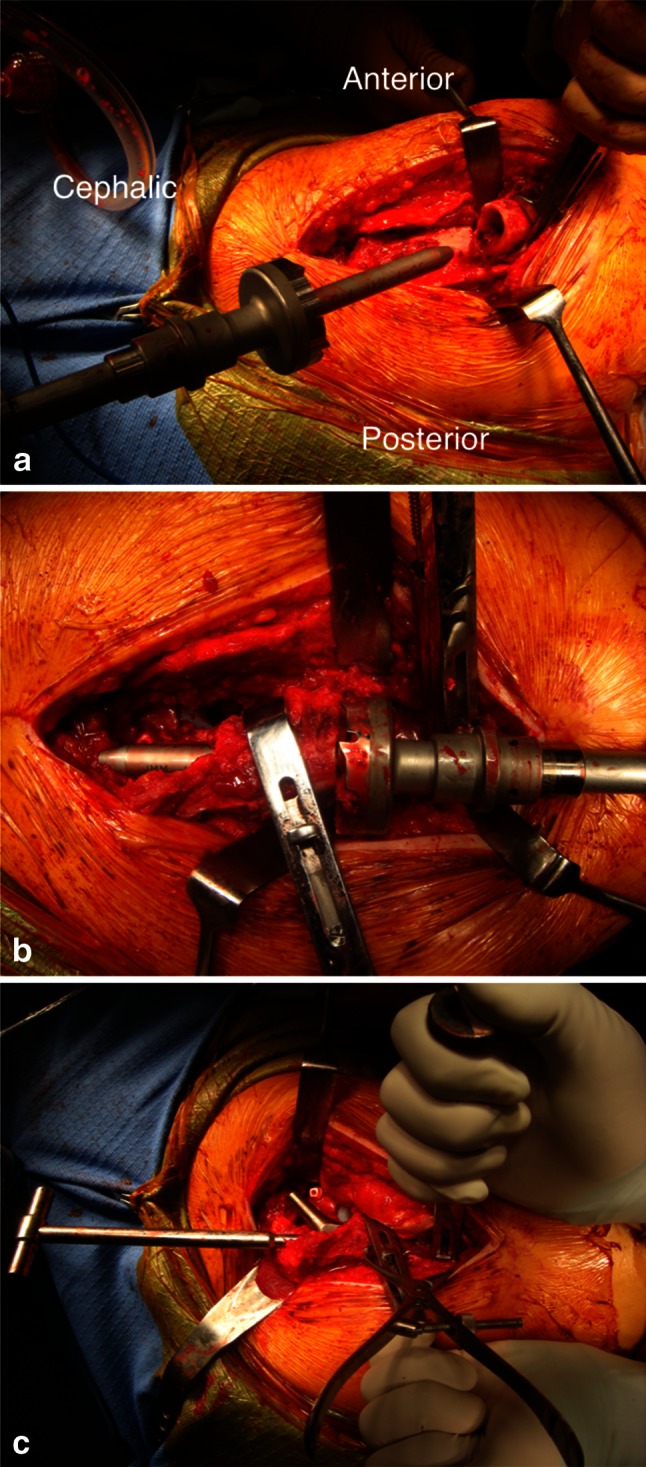
These photographs show **a** the planarization of the proximal cortical bone in the distal shaft, using the original device. **b** the planarization of the distal cortical bone in the proximal fragment, using the same device. **c** the bone holders that allow one assistant to firmly maintain the hip position during surgery

Next, the proximal femoral fragment was manually pulled distally, and muscle excursion was confirmed. Additional resection of the femur was performed, if necessary. After both the proximal fragment and the distal shaft of the femur were reamed and broached, the contact face in the transverse osteotomy, i.e., both proximal and distal cortical bones, was planarized using the original device for conformity (Fig. 3a, b). The trial stem was reduced to the acetabular component, after which we tested for leg length discrepancy, range of movement (ROM), impingement, and sciatic nerve tension. The femoral rotational position in the transverse osteotomy was marked on both the proximal fragment and the distal shaft of the femur using either a marking pen or an electrocautery scalpel. A surgical assistant used bone holders to keep both the proximal fragment and the distal shaft of the femur firmly in place (Fig. 3c).

After inserting the bone plug, we thoroughly irrigated the femoral canal and dried it with gauze. Vacu-Mix Plus ENDURANCE Bone Cement (DePuy International) was retrogradely inserted, using a cement gun, after which an HS32 N narrow stem (KYOCERA Medical) was implanted. We attempted to remove as much excess cement as possible by applying pressure to the stem and using a spatula to scrape away the cement that leaked from the osteotomy. We augmented the strut onlay autografts of the resected femoral shaft bone using Ethybond (Johnson & Johnson K.K., Tokyo, Japan) cerclage in the transverse osteotomy site. Once the hip reduction was complete, we directly palpated the sciatic nerve in neutral and flexed hip positions, and confirmed it to be free of tension; intraoperative continuous neurologic monitoring was not performed. Postoperatively, intravenous injection of 1.0 g cefazolin, every 12 h, was administered for 2 days; full weight bearing was not advised for the first 5 days, similar to conventional THA.

### Postoperative follow-up

After the surgery, the patients were followed-up at 2 weeks, 3, 6 months, and then annually; the mean follow-up period was 5.2 years (3–10 years), and none of the patients was lost to follow-up. The Japanese Orthopaedic Association (JOA) hip score [18] was used to clinically assess all of the hips. The JOA hip score consists of scores for pain (40 points), ROM (20 points), walking (20 points), and activities of daily living (20 points), with a maximum possible score of 100 points. We also looked for signs of surgical complications, including nerve injury, dislocation, and infection.

Blinded evaluations of AP radiographs were performed. At 2 weeks after surgery, the abduction angle and the height from the teardrop line to the center of the acetabular component were measured, and the presence of demarcation was determined on the acetabular side. On the femoral side, the lengthening of the tip of the greater trochanter was calculated by subtracting the postoperative height of the greater trochanter from the preoperative height; the height of the greater trochanter was measured as the vertical distance between the teardrop line and the tip of the greater trochanter. Cement interdigitation was evaluated by the classification of Mulroy et al. [19]. During follow-up examinations, we assessed the bone union at the osteotomy site and looked for evidence of loosening. The presence of radiolucent lines was evaluated according to DeLee and Charnley [20] for the acetabulum and according to Gruen et al. [21] for the femur; component loosening was defined according to the criteria of Hodgkinson et al. [22] and Harris et al. [23]. The spina malleolar distance was used as an indicator of leg lengthening, and was measured as the distance between the anterior superior iliac spine and the medial malleolus in the AP radiograph of the whole lower leg [24].

Hips were classified into one of five groups depending on the preoperative hip flexion angle, as follows: group I, 0°–30°; group II, 31°–60°; group III, 61°–90°; group IV, 91°–120°, group V, >121°. This allowed us to investigate the relationship between preoperative hip flexion and leg lengthening.

## Results

None of the hips required a revision of the acetabular or femoral components for any reason. JOA hip scores improved from a mean of 50.2 points (16–74 points) prior to surgery to an average of 84.6 points (62–97 points) at the latest follow-up. Hip flexion improved from a mean of 93.1° (40°–130°), prior to surgery, to an average of 93.6° (60°–120°) at the latest follow-up. There were three cases of dislocations. These were temporary and occurred within 1 month of the operation and were conservatively treated. Other complications, such as nerve injury or infection, were not observed.

On the acetabular side, the mean abduction cup angle was 44.3° (35°–50°) and the mean cup height was 19.1 mm (7–33 mm). Demarcation of the acetabular component was not observed at 2 weeks after surgery and there was no evidence of radiolucent lines or loosening seen during the most recent follow-up assessment. On the femoral side, the mean lengthening of the tip of the greater trochanter was 76.5 mm (25–120 mm). According to Mulroy’s classification, 16 hips were categorized as grade A, 13 hips as grade B, three hips as grade C1, and 2 hips as grade C2. All C1 and C2 hips had radiolucencies in zone 2 and zones 2–4, respectively. Progressive radiolucency at the osteotomy site was only observed in one hip; this was the only hip in which bone union was not achieved at the osteotomy site. The completion of bone union required an average of 7.7 months (3–24 months) in the remaining 33 THAs; radiographic evidence of loosening was not observed.

**Fig. 4:**
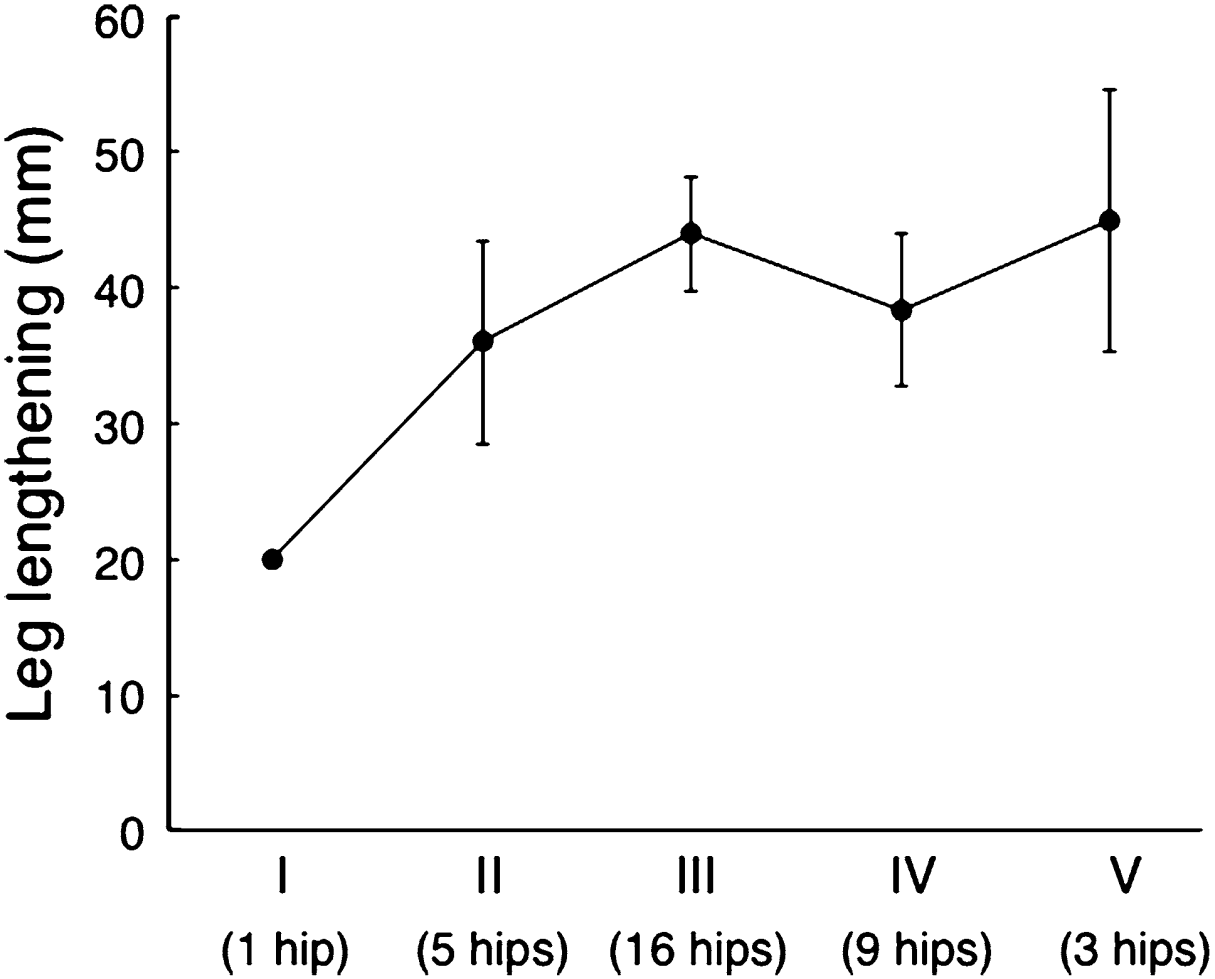
This graph shows the relationship between hip flexion and leg lengthening. Hips were classified into groups depending on their preoperative hip flexion angle, as follows: group I, 0°–30°; group II, 31°–60°; group III, 61°–90°; group IV, 91°–120°, and group V, >121°. Leg lengthening was measured using the spina malleolar distance in the anteroposterior radiograph of the whole lower leg

The mean leg lengthening was 40.5 mm (15–70 mm), and 42.0 mm (20–60 mm) in the eight patients who had undergone previous surgery. In the eight patients with unilateral dysplasia, the leg length discrepancy improved from a mean of 47.0 mm (35–50 mm) prior to the surgery to 12.2 mm (0–20 mm) after the procedure. In the 18 patients with bilateral dysplasia, the discrepancy improved from a mean of 28.4 mm (0–60 mm) to 7.0 mm (0–15 mm). The final leg length discrepancy was <10 mm in 16 patients and 10–20 mm in ten patients; none of the patients had a leg length discrepancy >20 mm. Based on preoperative hip flexion angle, we classified one hip as group I, five hips as group II, 16 hips as group III, nine hips as group IV, and three hips as group V. These were associated with mean leg lengthenings of 20.0, 36.0, 43.9, 38.3, and 45.0 mm, respectively (Fig. 4).

## Discussion

Although cemented THAs have great advantages because the femur is generally hypoplastic with a narrow and deformed canal, there are few literature reports regarding this procedure. Here, we have shown that severely dislocated hips can be effectively treated with cemented THA, combined with SSO.

There are some limitations in this study. First, the study was small, involving only 34 individuals. However, the indication for this procedure is relatively uncommon and obtaining a larger series from a single institution is difficult [10]. Second, our follow-up was limited to a minimum of 3 years. We have performed cemented THAs in patients of any age because we believe in the long-term durability. Currently, our philosophy may not be common and continued follow-up will be required to establish the long-term results of this procedure. Third, we could not accurately describe the amount of the femoral resection, because, after the resection of the femur, according to proper preoperative planning, additional resection of the femur was performed to allow adequate muscle excursion. Further, cortical bones were planarized for conformity between the proximal fragment and the distal shaft. Therefore, the amount of the femoral resection was calculated by subtracting the leg lengthening from the lengthening of the tip of the greater trochanter. Fourth, the spina malleolar distance was used as an indicator of leg lengthening, and was measured as the distance between the anterior superior iliac spine and the medial malleolus in the AP radiograph of the whole lower leg. This may not be the best way because the pelvic tilt could significantly influence this measurement. Fifth, because none of our patients experienced nerve injury, we were unable to directly investigate the relationship between this complication and the leg lengthening procedure.

**Fig. 5:**
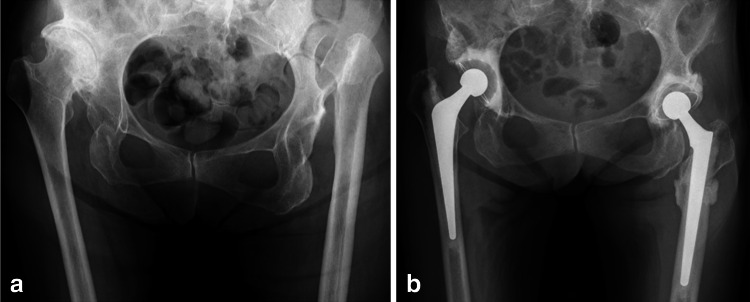
**a** A preoperative radiograph of the hips of an 80-year-old female showing bilateral dysplasia of the hip, classified as Crowe group IV. The hip flexion was 30° on the *right* side and 90° on the *left* side. **b** A radiograph taken 3 years after the surgery shows bone union at the osteotomy site and no evidence of loosening. Total hip arthroplasty (*THA*) with subtrochanteric shortening osteotomy was performed on the *left side*, but conventional THA was performed on the *right hip* because of limited elongation

Currently, many authors advocate the use of SSO for severely dislocated hips. However, Kawai et al. [11] reported that, in 41 THAs in Crowe group IV, 19 (46 %) hips required SSO, and Kerboull et al. [7] reported that, in 118 THAs in Crowe group IV, only two hips (2 %) needed to be combined with this procedure. These findings suggest that this procedure is not essential for severely dislocated hips. Conventional THAs are safer and easier, and should, therefore, be performed whenever possible—even when hips have been classified as Crowe group IV; a thorough preoperative assessment is necessary to determine an appropriate surgical course. We have found that AP radiographs are particularly useful during preoperative assessments and we recommend that these be taken not only in a neutral hip position but also in a flexed adduction position (Fig. 2). This allows evaluation of the expected descent of the tip of the greater trochanter. Such an evaluation is important to provide the information necessary for preserving the muscle excursion of the gluteus medius and the external rotator, and to maximize postoperative function. We suggest the use of SSO in situations where the greater trochanter height is markedly different in the neutral and flexed adduction hip positions. Conversely, when the height differs little, i.e., with a new false acetabulum, we suggest compromising on the position of the acetabular component, since SSO will have little effect (Fig. 5).

While some authors have recommended limiting leg lengthening to 40 mm [3, 25–28], others have suggested that it is possible to lengthen legs by as much as 70 mm [7, 29, 30]. In a study of 508 THAs for DDH, Eggli et al. [31] found no statistically significant correlation between leg lengthening and nerve injury. Similarly, we found that leg lengthening did not lead to nerve injuries in any of our patients. Interestingly, we also found that leg lengthening was greater in patients without hip flexion contracture than in those with it (Fig. 4), even though muscle release was not required for leg lengthening because of the degree of elongation. These observations denote the same tendency, regardless of past surgeries. They also demonstrate that preoperative assessment of the contracture is essential. However, there is no safe method of leg lengthening in THA for a severely dislocated hip, and it is recommended to make additional femoral cuts in cases requiring leg lengthenings >40 mm, with intraoperative assessment of nerve palpation.

Based on these results, we have three main recommendations for successful surgery. First, in cases of SSO for severely dislocated hips, we advocate cemented THA because implants with a straight and narrow stem are anatomically suitable for patients with severely dislocated hips. For such an abnormal configuration of the femoral canal, a cemented THA has great advantages with respect to the ease-of-use handling of the medullary canal. In previous reports of relatively large series, including more than 15 cemented THAs, there were few revisions for aseptic femoral loosening over a minimum follow-up period of 0.5–4.3 years [10–13] (Table 2). Similarly, radiographic evidence of loosening was not observed in the current study. Second, we advise surgeons to perform transverse osteotomies [5], which facilitate correction of abnormal configurations and the removal of cement from the osteotomy site. The aim of this step is to allow adequate reduction of the hip, accurately restore the rotational alignment of the proximal femur, and improve the lever arm of the abductor musculature by restoring the lateral position of the greater trochanter [12]. Transverse osteotomies can easily correct excessive anteversion or other angular deformities that are commonly observed in severely dislocated hips. Several other osteotomy techniques are available, including step cut [32], double-chevron [33], and oblique [34]; all of these can help achieve rigid initial fixation when employed with press-fit stems. However, a cemented THA is superior to initial fixation even if transverse osteotomy is used. The only major disadvantage of a transverse osteotomy, relative to the other techniques, is that there is a smaller bone contact area, which may delay bone union. Regardless, previous authors have reported that the use of onlay autografts can increase the success of THAs involving a transverse osteotomy [11, 35]. Third, we suggest the use of original devices to achieve conformity and bone holders to maintain bone position (Fig. 3). Cumulatively, these techniques will allow surgeons to achieve both rigid initial fixation and secure bone unions.

**Table 2:** Recent published studies reporting on relatively large series of cemented THA combined with subtrochanteric shortening osteotomy

Study	Year published	Number	Mean follow-up in years (range)	Bone union (%)	Revision for aseptic femoral loosening (%)
Howie et al. [10]	2010	35	5.6 (2.0–14.0)	33 (94)	3 (9)
Kawai et al. [11]	2011	19	3.5 (0.5–8.0)	19 (100)	0
Charity et al. [12]	2011	18	9.5 (4.3–14.0)	17 (94)	0
Akiyama et al. [13]	2011	15	6.3 (2.8–10.4)	12 (80)	0
Current study	2013	34	5.2 (3.0–10.0)	33 (97)	0

## Conclusions

SSO combined with cemented THA is a safe and reliable procedure for restoring the anatomic hip center and trochanteric rotation. Our results indicate that nerve injury can be avoided even when leg lengthening occurs; this may be related to the lack of contracture in severely dislocated hips. Because there is no completely safe leg lengthening method in THA for a severely dislocated hip, it is important to consider preoperative ROM and also to perform intraoperative nerve palpation.
